# Dendritic cells: key players in human herpesvirus 8 infection and pathogenesis

**DOI:** 10.3389/fmicb.2014.00452

**Published:** 2014-08-28

**Authors:** Diana M. Campbell, Giovanna Rappocciolo, Frank J. Jenkins, Charles R. Rinaldo

**Affiliations:** ^1^Department of Infectious Diseases and Microbiology, Graduate School of Public Health, University of PittsburghPittsburgh, PA, USA; ^2^Department of Pathology, School of Medicine, University of PittsburghPittsburgh, PA, USA

**Keywords:** human herpesvirus 8 (HHV-8), herpesviruses, dendritic cells, Kaposi’s sarcoma-associated herpesvirus (KSHV), Kaposi’s sarcoma, primary effusion lymphoma, multicentric Castleman’s disease, DC-SIGN

## Abstract

Human herpesvirus 8 (HHV-8; Kaposi’s sarcoma-associated herpesvirus) is an oncogenic gammaherpesvirus that primarily infects cells of the immune and vascular systems. HHV-8 interacts with and targets professional antigen presenting cells and influences their function. Infection alters the maturation, antigen presentation, and immune activation capabilities of certain dendritic cells (DC) despite non-robust lytic replication in these cells. DC sustains a low level of antiviral functionality during HHV-8 infection *in vitro.* This may explain the ability of healthy individuals to effectively control this virus without disease. Following an immune compromising event, such as organ transplantation or human immunodeficiency virus type 1 infection, a reduced cellular antiviral response against HHV-8 compounded with skewed DC cytokine production and antigen presentation likely contributes to the development of HHV-8 associated diseases, i.e., Kaposi’s sarcoma and certain B cell lymphomas. In this review we focus on the role of DC in the establishment of HHV-8 primary and latent infection, the functional state of DC during HHV-8 infection, and the current understanding of the factors influencing virus-DC interactions in the context of HHV-8-associated disease.

## BACKGROUND

Human herpesvirus 8 (HHV-8) is one of eight herpesviruses known to infect and establish chronic latency in humans. Also known as Kaposi’s sarcoma-associated herpesvirus (KSHV), HHV-8 was first identified as a gammaherpesvirus in 1994 in AIDS patients with Kaposi’s sarcoma (KS; Chang et al., 1994). HHV-8 has since been recognized as the causal agent of all four distinct forms of KS: classic KS in elderly European and Mediterranean men, endemic KS in human immunodeficiency virus type 1 (HIV-1) negative African populations, iatrogenic KS in immunosuppressed patients post-transplantation, and AIDS-KS in HIV-1 infected populations. It is also acknowledged as a common factor in the development of two B cell lymphomas, i.e., primary effusion lymphoma (PEL) and a subset of Castleman’s disease cases known as multicentric Castleman’s disease (MCD; Chang et al., 1994; Cesarman et al., 1995; Soulier et al., 1995; Nador et al., 1996; Cesarman and Knowles, 1999).

This double-stranded DNA virus targets several cell types for infection *in vivo* and *in vitro* where it either replicates or establishes latency. Of particular interest is that HHV-8 targets so-called professional antigen presenting cells (APC) including monocyte-derived dendritic cells (MDDC; Rappocciolo et al., 2006a), B cells (Ambroziak et al., 1995; Rappocciolo et al., 2008; Myoung and Ganem, 2011) and monocytes (Blasig et al., 1997), as well as endothelial cells (Boshoff et al., 1995). Indeed, while HHV-8 infected and uninfected endothelial and spindle cells form neoplastic KS lesions (Boshoff et al., 1995), infected and uninfected B cells and monocytes are found proximal to KS lesions (Ambroziak et al., 1995; Blasig et al., 1997). The role of dendritic cells (DC) in antigen presentation and immune activation suggests these functions, or lack thereof, could be involved in the development of HHV-8 diseases associated with a compromised immune system.

Myeloid dendritic cells (mDC), including Langerhan cells (LC), skin dermal dendritic cells (DDC), submucosal dermal dendritic cells (SMDC), and MDDC, have essential roles in both the innate and adaptive immune response to primary and subsequent infections as well as reactivation of chronic viral infections. They act during the innate immune response as specialized cells that survey and detect antigens of foreign microorganisms throughout the body thereby inducing their ability to communicate with helper and effector lymphocytes to bridge the innate and adaptive response (Clark et al., 2000; Geissmann et al., 2010). In addition, plasmacytoid dendritic cells (pDC) of lymphoid origin recognize single-stranded RNA and unmethylated CpG motifs associated with viral infection to help induce an antiviral response within the host (West et al., 2011). Infection of monocytes and DC by both gammaherpesviruses, HHV-8 and Epstein–Barr virus (EBV), has been shown to diminish the subsequent T cell response (Li et al., 2002; Rappocciolo et al., 2006a; Hensler et al., 2009). However, the manner in which DC infection alters the cellular response associated with HHV-8 infection and its impact on HHV-8 associated disease is minimally understood.

Despite multiple attempts to generate reliable animal models of HHV-8-associated diseases, the overall lack of suitable *in vivo* models has limited HHV-8 pathogenesis research. A humanized-BLT mouse model has recently been successfully used to establish lytic and latent infection in human B cells and macrophages (Wang et al., 2014), and augurs well for future research on HHV-8. Here we focus on the role of DC in the establishment of HHV-8 primary and latent infection predominantly using *in vitro* models, including the functional state of DC during HHV-8 infection and the current understanding of the factors influencing virus – DC interactions in the context of HHV-8-associated disease.

## CELLULAR RECEPTORS FOR HHV-8 INFECTION

### ATTACHMENT RECEPTORS

The establishment of HHV-8 infection requires two separate events at the surface of susceptible cells – namely binding to an attachment receptor followed by binding to one or more entry receptors. Prior to the initiation of virus entry, attachment occurs by the direct interactions of viral glycoproteins B (gB) and K8.1 with the attachment receptor heparan sulfate (HS) on the cell surface (Akula et al., 2001a,b; Birkmann et al., 2001; Wang et al., 2001a). This has been supported by evidence that soluble heparin, a molecule similar in structure to HS, blocks HHV-8 attachment to fibroblasts in a dose-dependent manner (Akula et al., 2001b). However, soluble heparin is not sufficient to completely block HHV-8 infection of fibroblasts or endothelial cells (Akula et al., 2001b; Birkmann et al., 2001), suggesting that virus entry is a multi-step process and there may be other attachment factors involved. Yet, the ubiquitous nature of HS expression on host cells may explain the broad range of cellular targets of this virus.

### ENTRY RECEPTORS

Several receptors in the extracellular matrix have been implicated in HHV-8 entry of different human cell types. Dendritic cell-specific ICAM-3-grabbing non-integrin (DC-SIGN) has been revealed as a receptor for HHV-8 infection of MDDC, monocytes, and monocyte-derived macrophages (Rappocciolo et al., 2006a; Kerur et al., 2010), as well as B cells (Rappocciolo et al., 2008). More recently we have shown that gB of HHV-8 binds to DC-SIGN (Hensler et al., 2014). In addition, our group has shown that HHV-8 infection of MDDC is associated with a decrease in DC-SIGN expression (Rappocciolo et al., 2006a). Integrin α_3_β_1_ has been demonstrated as a cellular receptor for HHV-8 infection on vascular endothelial cells and foreskin fibroblasts (Akula et al., 2002). Most recently, the ephrin receptor tyrosine kinase A2 has been found to serve as a receptor for HHV-8 infection mediated by interactions with the viral glycoprotein L and H dimer, specifically on HS-negative cells (Hahn et al., 2012), which had previously been identified as a viral surface complex essential for entry into susceptible host cells (Campadelli-Fiume et al., 2007). Interestingly, this receptor has been shown to be expressed on LC (Munthe et al., 2004), suggesting that these cells could be susceptible to HHV-8 infection.

These reports support that HHV-8 is capable of recognizing multiple receptors on human host cells, and emphasize that our understanding of HHV-8 attachment, entry, and possibly spread throughout the host is still limited. Minimal data exist demonstrating the role of gL and gH in HHV-8 infection, specifically on HS-positive cells that likely use HS as an initial receptor for attachment. The viral gB has been demonstrated to bind to both DC-SIGN and the integrin α_3_β_1_ (Akula et al., 2002; Sharma-Walia et al., 2004). It is unclear whether either receptor alone is sufficient for both viral attachment and entry. HHV-8 (like other human herpesviruses) likely utilizes receptor flexibility to expand the range of cell types it infects and adapt to different immunological environments that it encounters in the host.

## HHV-8 TRANSMISSION

### MUCOSAL TRANSMISSION

The oral cavity is the most common portal of entry and route of transmission for HHV-8 (Pica and Volpi, 2007) leading to inevitable virus exposure to mucosal surfaces of the oropharynx. In fact, HHV-8 DNA can be detected by PCR from intact virions in the majority of saliva samples from seropositive individuals (Koelle et al., 1997; Vieira et al., 1997). Given the strategic positions of LC and SMDC at the mucosal sites, these cells could be the first targets of HHV-8 infection. The primary role of such mDC is the detection, uptake, and processing of foreign microbes for antigen presentation to T cells in secondary lymphoid organs, an efficient method of activating the adaptive immune response and establishing immunologic memory against invaders. Based on this function of mDC, they are constantly exposed to and interacting with pathogens and are thereby likely targets of HHV-8 infection. LC are the primary subset of DC found in the epidermal layer of the skin and within the epithelia lining mucosal surfaces (Iwasaki, 2007), both of which are functional physical barriers that serve as the primary defense mechanism against pathogens. Although LC do not express the HHV-8 entry receptor DC-SIGN (Soilleux and Coleman, 2001; Soilleux et al., 2002), they do express the HHV-8 attachment receptor HS on their surface (Yan et al., 2004). LC also express langerin, a type II transmembrane C-type lectin which could potentially be also acting as a receptor for HHV-8 (Valladeau et al., 2000). Interestingly, langerin has been shown to bind HIV-1 and subsequently transport it to the Birbeck granules for degradation, thus forming a protective barrier to HIV-1 dissemination (de Witte et al., 2007). Such studies of LC in HHV-8 infection are hampered by difficulties in obtaining human LC in sufficient quantities and a natural homeostatic state. Alternatively, susceptibility to HHV-8 infection could be assessed using an established *in vitro* model of deriving LC from CD34^+^ hematopoietic cells (Ratzinger et al., 2004). Interestingly, infection of LC plays a role in the pathogenesis of EBV (Walling et al., 2007). Whether LC are targets for HHV-8 infection, and langerin acts as a receptor or protective factor, may open a new avenue toward understanding HHV-8 establishment of latency and reactivation.

In addition to LC, SMDC are found in lamina propria below the epithelial surface of the oral cavity (Cutler and Jotwani, 2006). *In vitro* and *in vivo* HHV-8 infection of SMDC has yet to be shown. Nonetheless, unlike LC, SMDC express DC-SIGN (Cutler and Jotwani, 2006) suggesting that they are permissive to HHV-8 infection (Rappocciolo et al., 2006a). In conclusion, due to their physical location and central function in T cell activation, it would be physiologically relevant for HHV-8 to target mucosal residing DC for replication and transport into the lymphatic system.

Human herpesvirus 8 transmission through the genital tract is considered less likely due to the lower levels of detectable virus found in semen and vaginal secretions compared to saliva (Edelman, 2005). However, it is still a major route of transmission and of great concern in high-risk endemic communities (Pica and Volpi, 2007). Interestingly, HHV-8 has been detected in tissue of normal non-cancerous prostates from men who have succumbed to AIDS (Montgomery et al., 2006). HHV-8 can also be detected in cervicovaginal secretions of HIV-1/HHV-8 co-infected women (Lisco et al., 2006), and although it is rare, vertical transmission of HHV-8 has been recorded (Brayfield et al., 2003). LC and SMDC are both found within the epithelial lining of the ectocervix and the vagina (Iwasaki, 2007). The function of immune cells in the female reproductive tract is known to be heavily controlled by hormone production, and their phenotype and function is not fully understood (Iwasaki, 2007). Therefore, based on possible differences in receptor expression, the susceptibility of genital tract DC to HHV-8 infection may vary compared to their counterparts in oral mucosa, which could at least in part explain the differential transmission seen between oral and sexual routes.

### BLOOD-BORNE TRANSMISSION

Human herpesvirus 8 can be transmitted through exposure to infectious blood products. Cases of HHV-8 seroconversion associated with blood transfusion, organ transplantation, and intravenous drug use have been reported, however, the latter may be indicative of other high-risk behaviors associated with drug use (Pica and Volpi, 2007). Blood-borne transmission directly exposes immune cells circulating in the vascular system, such as mDC, to the virus much quicker than natural exposure through the oral or genital route. mDC are sparsely distributed throughout the body (Banchereau and Steinman, 1998), which has made them difficult to study. The development of an *in vitro* model of deriving MDDC from peripheral blood monocytes with GM-CSF and interleukin 4 (IL-4; Sallusto and Lanzavecchia, 1994) eased the study of this DC subpopulation, as they closely resemble the function of *in vivo* mDC (Qu et al., 2009; Cheong et al., 2010). The *in vitro* derived MDDC express DC-SIGN on their surface through which HHV-8 enters and establishes low levels of lytic replication, but display little viral DNA turnover (Rappocciolo et al., 2006a). Differences in primary sites of HHV-8 infection likely influence the host response to infection. Due to a non-robust replication cycle within MDDC and little evidence that LC and SMDC support HHV-8 replication, it is likely the virus also spreads further into the host through cells other than DC.

## HHV-8 INFILTRATION OF THE LYMPHATIC SYSTEM

### TRAFFICKING TO LYMPHOID ORGANS

How HHV-8 travels to and infects cells within secondary lymphoid tissue is not fully understood. The mucosal immune system, both oral and urogenital, is highly sensitized to the detection of and protection from foreign microbes. However, when an organism breaches this primary line of defense, resident immune cells detect the intrusion and produce pro-inflammatory cytokines. Such soluble molecules increase the migration of maturing LC and SMDC into the mucosal lamina propria (Jotwani and Cutler, 2003) where they engulf foreign antigen, upon which lymphatic homing receptors are up-regulated, such as CCR7, to attract them to CCL19 and CCL21 expressing high endothelial venules (HEVs). This triggers APC migration into lymphatic vessels and trafficking to draining lymph nodes where the foreign antigen is presented to effector T and B cells (Holmgren and Czerkinsky, 2005).

Human herpesvirus 8 likely travels to draining lymphoid organs within DC and monocytes from the primary site of infection, where it can then interact with resident B and T cells. Lytic HHV-8 infection of oral epithelium occurs *in vitro* (Duus et al., 2004), and has been detected *in vivo* in monocytes within the cutaneous, highly vascularized lesions of KS patients (Blasig et al., 1997). Macrophages found in tissues and monocytes in peripheral blood travel to draining lymph nodes upon stimulation by foreign microbial antigens, and function in antigen presentation. Macrophages treated with IL-13 express DC-SIGN (Chehimi et al., 2003), resulting in their susceptibility to abortive infection with HHV-8 (Rappocciolo et al., 2006a). Moreover, tissue resident macrophages express DC-SIGN (Soilleux et al., 2002; Granelli-Piperno et al., 2005). Therefore, it can be hypothesized that HHV-8 infects the mucosal epithelium and uses proximal DC, and possibly monocytes and macrophages, to penetrate the lymphatic system.

### INTERACTION WITH B CELLS

Immature B cells are abundant in the germinal centers of lymph nodes where they interact with antigen presenting follicular DC (fDC) and follicular T helper cells, and mature during somatic hypermutation and clonal expansion (Beltman et al., 2011). B cells are known to harbor HHV-8 in individuals with KS, PEL, and MCD (Ambroziak et al., 1995; Cesarman et al., 1995; Cesarman and Knowles, 1999). In fact, HHV-8 isolated from PEL-derived cell lines has been used to infect CD19^+^ B cells *in vitro* (Mesri et al., 1996; Rappocciolo et al., 2008). Extensive evidence indicates that peripheral blood B cells from healthy individuals support productive lytic HHV-8 infection *in vitro* when activated with CD40L and IL-4, whereas tonsillar B cells do so without requiring *ex vivo* activation (Rappocciolo et al., 2008). More recent work in our laboratory shows that HHV-8 targets a range of naïve B cell, IgM memory B cell, and plasma cell-like populations both after *in vitro* infection and circulating in the blood of HIV-1 infected persons with KS (Knowlton et al., 2014).

B cells could also serve as a reservoir of latent HHV-8, as it has been reported that the latency-associated nuclear antigen (LANA-1) can be detected in IgM^+^ B cells of tonsil origin (Hassman et al., 2011). It is unclear whether each of these B cell subsets is directly infected or derived from infected precursors. Interestingly, CD4^+^ T cells are suggested to support latent HHV-8 infection in B cells by reducing the viral lytic cycle (Myoung and Ganem, 2011). Finally, HHV-8 DNA is detectable in B cells of people with PEL (Cesarman et al., 1995), MCD (Dupin et al., 1999), and KS (Huang et al., 1997; Knowlton et al., 2014). Studies are needed to more directly assess the role of HHV-8 infection of B cells in endothelial cell transformation associated with KS.

Our most recent data show that HHV-8 infected B cells display a novel polyfunctional cytokine production profile, with an abundance of IL-6, tumor necrosis factor α (TNFα), macrophage inhibitory protein-1α (MIP-1α), macrophage inhibitory protein-1β (MIP-1b) and IL-8 after *in vitro* infection and in the blood of HHV-8/HIV-1 co-infected subjects with KS (Knowlton et al., 2014). This systemic, B cell cytokine and chemokine milieu could be important in driving endothelial cell outgrowth in KS, and contribute to the development of HHV-8 associated malignancies. However, it is still unclear whether various B cell lineage subsets are equally susceptible to HHV-8 lytic and latent infection, or if B cell subset precursors are infected and as a result driven into differentiation. Investigation of the natural progression of HHV-8 infection in B cells, as well as DC and monocytes/macrophages, is required to bridge the gap in our knowledge between the APC viral reservoir and transformation of endothelial cells in KS.

### INTERACTION WITH T CELLS

Naïve T cells enter lymph nodes through HEVs where they interact with incoming APC, with both cell types concentrating in paracortical zones where T cell activation occurs (Liao and Padera, 2013). The killing function of cytotoxic CD8^+^ T lymphocytes (CTLs) in response to intracellular viral antigen presentation is paramount to clearing the body of infected cells. MHC class I restricted (CD8^+^) responses to HHV-8 protein specific epitopes have been detected (Osman et al., 1999; Wang et al., 2002; Wilkinson et al., 2002). HHV-8 specific T cell responses in individuals are not as pronounced as, for example, EBV (Hislop et al., 2007; Robey et al., 2010), but are nonetheless sufficient to control primary HHV-8 infection and provide life-long protection from HHV-8 cancers in immunocompetent individuals (Wang et al., 2001b).

Follicular DC make up a subset of DC with ontogeny distinct from mDC and pDC that reside in secondary lymphoid organs and specialize in antigen presentation (King and Katz, 1990) as well as provide anti-apoptotic stimuli during activation of B cells (Park and Choi, 2005). HHV-8 is not detectable, based on monoclonal antibody staining for ORF73 (LANA-1), in fDC within lymph nodes isolated from KS, PEL, and MCD patients (Dupin et al., 1999). However, a more comprehensive and sensitive analysis of lytic and latent gene expression using an established DNA array of every HHV-8 ORF (Jenner et al., 2001) may be more insightful for detecting HHV-8 infection of not only fDC but other target cells. It is important to further investigate if fDC interactions with T and B cells influence HHV-8 infection and pathogenesis.

Previous studies by our group demonstrate that HHV-8 infection of MDDC *in vitro* reduces their level of endocytosis as well as inhibits their ability to activate virus-specific CD8^+^ T cells (Rappocciolo et al., 2006a). In addition, Gregory et al. (2012) established latent HHV-8 infection of a monocyte cell line *in vitro* as a model to study the impact of HHV-8 on monocyte function. Latent viral infection of the monocyte cell line was associated with a dampened inflammatory and adaptive immune response, indicated by a decrease in proinflammatory cytokine production and monocyte co-stimulatory molecule expression associated with T cell activation. It has also been reported that HHV-8 can enter T lymphocytes from tonsils and result in an abortive infection (Myoung and Ganem, 2011). Finally, LC are known to strongly induce activation of CD4^+^ (Furio et al., 2010) and CD8^+^ T cells (Klechevsky et al., 2008), opening the possibility that HHV-8 directly affects this ability of LC to activate T cells. In sum, whether virus is interacting with MDDC and monocytes in circulation or tissue, or with LC and SMDC in the mucosa, T cell responses are likely altered. This must be viewed however, with the fact that HHV-8 infection does not result in high-risk for disease in immunocompetent people.

## DC IN HHV-8-ASSOCIATED DISEASE

Despite the wide range of permissive and potentially susceptible host cells and substantial immune modulation associated with infection, most HHV-8-associated pathologies only occur when the immune response is compromised by HIV-1 co-infection or another secondary mechanism such as immunosuppression caused by anti-rejection drugs during organ transplantation and by older age. AIDS-associated cancers, including KS and non-Hodgkin’s lymphomas such as PEL are strongly influenced by T cell immunosuppression (Frisch et al., 2001). In addition, KS has been shown to develop more quickly if primary HHV-8 infection occurs after HIV-1 infection (Jacobson et al., 2000). Based on such epidemiological data, substandard T cell function and consequent ineffective control of virus lytic reactivation are a major basis for susceptibility to HHV-8 disease. This potentially implicates abnormalities in DC as a basis for such T cell dysfunction during HHV-8 infection.

### KAPOSI’S SARCOMA

Human herpesvirus 8 was initially isolated from tissue samples of AIDS-associated KS patients and identified as a unique gammaherpesvirus similar to but distinct from EBV (Chang et al., 1994). Today, HHV-8 is associated with all forms of KS irrespective of immunocompromised status. Perhaps what is most puzzling is that the extensive array of lytic and latent gene products encoded by HHV-8 that mimic host immunoregulatory proteins as well as growth factors are insufficient to cause KS (Mesri et al., 2010). We (Knowlton et al., 2012) and others (Rezaee et al., 2006) hypothesize that a specific cytokine and chemokine environment enhances outgrowth of HHV-8 infected, vascular endothelial cells. It is therefore likely that the impact of HHV-8 infection of DC on T and B cell cytokine and chemokine production is important in this pathogenic process.

From a historical perspective, DDC were associated with both AIDS-associated and non-AIDS-associated KS lesions prior to the identification of HHV-8 as the etiological agent of KS (Nickoloff and Griffiths, 1989), yet data concerning DDC in AIDS-associated KS are limited and need to be expanded. In addition, multiple cell types associated with the dermal and epidermal layers in KS lesions support their role in productive HHV-8 infection. Thus, HHV-8 DNA has been detected in spindle cells and endothelial cells within and overlaying KS lesions (Nickoloff and Griffiths, 1989; Boshoff et al., 1995; Reed et al., 1998). DC quantity and functional quality are lowered or skewed in classic KS patients. These patients present with a lower frequency of MDDC and pDC, as well as an up-regulation of the endocytic receptor CD91 and decrease in expression of the T cell maturation factor IL-12 compared to HHV-8 positive individuals without KS (Della Bella et al., 2006). The likelihood of KS development and time to progression after HHV-8 seroconversion varies based on history of previous infections, HIV-1 co-infection, and behavioral characteristics (Armenian et al., 1993). In addition, highly active antiretroviral therapy (HAART) greatly extends the time to progression to AIDS (Hogg et al., 1999), and is associated with a decrease in HHV-8 replication and KS regression (Gill et al., 2002). Therefore, there may be events interfering with the anti-HHV-8 T cell response that begin years prior to disease. T cell responses to HHV-8 antigens and the role of DC in these have yet to be compared longitudinally from infection to KS development, and may reveal important implications of immune suppression and KS. There is a similar limited amount of *in vivo* data assessing T cells and DC in PEL and MCD.

### PRIMARY EFFUSION LYMPHOMA

Primary effusion lymphoma is an uncommon B cell lymphoma and comprises 4% of all HIV-1-related non-Hodgkin’s lymphoma cases (Simonelli et al., 2003). The presence of HHV-8 infection of oncogenic cells in HIV-1-related (Cesarman et al., 1995; Carbone et al., 1996b; Otsuki et al., 1996; Gessain et al., 1997) and non-HIV-1-related PEL (Nador et al., 1995; Carbone et al., 1996a; Said et al., 1996) is well established, however, the impact of infection on disease development remains unclear. Genome wide analysis of HHV-8 gene expression in latently infected PEL cell lines has demonstrated that multiple virally encoded products, including ORF71 and 72, v-FLIP, and v-cyclin, are expressed (Jenner et al., 2001), which could influence growth characteristics of the infected and uninfected cells.

As seen in KS, there is evidence that the functional quality and phenotype of DC are irregular in patients that present with PEL. Similarly, DC functional abnormalities have been associated with several cancers including melanoma and chronic lymphocytic leukemia (Enk et al., 1997; Orsini et al., 2003). Cirone et al. (2008) demonstrated a reduction in immature DC (iDC) differentiation from CD14^+^ monocytes, as well as phenotypic differences, in the presence of multiple individual PEL cell lines when co-cultured *in vitro,* compared to iDC derived in the absence of PEL cell lines. This suggests that in addition to reducing the function of DC, HHV-8 can inhibit the activation and differentiation of monocytes into DCs.

### MULTICENTRIC CASTLEMAN’S DISEASE

Multicentric Castleman’s disease is another rare lymphoproliferative disease highly correlated with HIV-1 infection (Oksenhendler et al., 1996) that occurs less commonly in HIV-1 seronegative individuals, the type of cases within which it was initially described (Castleman et al., 1956). Frequent co-presentation of MCD and KS raised questions regarding the relationship between MCD and HHV-8. During the same year HHV-8 was isolated from PEL cases, Soulier et al. (1995) detected HHV-8 in 100% of HIV-1-associated MCD cases and 41% of HIV-1 negative MCD cases. Interestingly, fDC sarcoma is a rare malignant neoplasm that has been associated with Castleman’s disease (Chan et al., 2001; Farah et al., 2005), but not yet MCD specifically. Small percentages of fDC from lymph node and spleen samples of MCD cases have been found to express HHV-8 LANA-1. Interestingly, these same fDC are thought to facilitate the increase of T cell infiltration into these secondary lymphoid organs (El-Daly et al., 2010). This suggests that fDC serve as functional soldiers in the DC arm of defense.

### MULTIPLE MYELOMA

Briefly, the design and use of DC-based treatment and vaccines targeting multiple myeloma was heavily considered and thought to be promising in the late 1990s. HHV-8 infection was initially found in bone marrow DC of patients with multiple myeloma (Rettig et al., 1997). However, evidence since has concluded that bone marrow-derived (Mitterer et al., 1998), peripheral blood mononuclear cell (PBMC)-derived (Cull et al., 1998; Yi et al., 1998), and CD34 positive cell-derived (Mitterer et al., 1998) DC from myeloma patients do not contain HHV-8 sequences. In fact, the absence of HHV-8 in DC of these patients increases the feasibility of an autologous, DC-based immunomodulatory therapy for multiple myeloma. However, such treatments have rarely been successful in clinical trials.

## EFFECT OF HHV-8 INFECTION ON DC

Human herpesvirus 8 encodes many cellular protein homologs, such as vIL-6 (Neipel et al., 1997), vMIP-I and -II (Moore et al., 1996), vIRF-1 (Lin et al., 2001), and vGPCR (Cesarman et al., 1996), that could dysregulate the host immune response and increase survival of latently infected cells (Jenner et al., 2001), thus playing a major role in HHV-8 pathogenesis. DC function is notably altered in KS, PEL, and MCD cases as previously described in this review, and the impact of direct HHV-8 infection of DC (Chan et al., 2001; Della Bella et al., 2006; Cirone et al., 2008) correlates with a dampening of DC functionality. A recent report suggests that one manner by which HHV-8 alters DC function is by blocking autophagy, a type of non-apoptotic programmed cell death, through STAT3 activation and the production of IL-6, IL-10, and IL-23 in MDDC *in vitro* (Santarelli et al., 2014). In addition, HHV-8 infection of DC precursor monocytes has been directly correlated with an inhibition of maturation into iDC and a decrease in functional stimulation of lymphocytes (Cirone et al., 2007).

A dysfunction in immune stimulation of infected DC is supported by earlier studies revealing two HHV-8 encoded proteins, K3 and K5, with essential roles in down-regulating MHC class I molecules via endocytosis from the surface of infected cells (Coscoy and Ganem, 2000; Ishido et al., 2000). Furthermore, despite sharing a 40% amino acid homology, structural differences between K3 and K5 are responsible for the specificity of the class I subtype targeted by each (Ishido et al., 2000). While K3 strongly down-regulates all four MHC class I types, K5 only targets HLA-A, B, and C, the latter of which is only weakly down-regulated (Ishido et al., 2000). Interestingly, it has been suggested that K3 and K5 have the ability to decrease levels of DC-SIGN and DC-SIGNR on the surface of virus-infected HEK293 kidney cells (Lang et al., 2013). HHV-8 encoded LANA1 inhibits MHC class I presentation on the surface of HEK293 (Kwun et al., 2011). However, it has yet to be revealed if LANA1 functions in this manner in DC. HHV-8 also produces multiple microRNAs, all of which are encoded within latency-associated genes (Samols et al., 2005), and are detectable in latently infected B cell lines suggesting that they play a role in inducing and/or maintenance of HHV-8 latency (Cai et al., 2005). However, their impact on DC function is not clear and could be studied with RNA silencing technology.

Despite an increased progression to disease following immune suppression, it is puzzling how otherwise healthy individuals remain capable of minimizing HHV-8 pathogenesis even with viral factors directly attempting to inhibit the function of DC and other immune cells. Addressing this enigma requires in depth studies of HHV-8 infection of naturally targeted cells including DC.

## FUNCTIONAL DC RESPONSES TO HHV-8 INFECTION

Despite seemingly extensive attempts by the virus to inhibit DC function and evade the host immune response, reports support that DC can contribute positively to the host’s battle against HHV-8 infection, as also described in innate and adaptive responses against EBV (Munz, 2014). Up-regulation of DC-SIGN expression on MDDC and B cells during maturation can be induced by IL-4 and CD40L (Relloso et al., 2002; Rappocciolo et al., 2006b). In contrast, type 1 anti-inflammatory cytokines such as interferon (IFN) and TGF-β trigger down regulation of DC-SIGN expression of MDDC (Relloso et al., 2002). Together these data suggest that DC-SIGN is positively regulated in response to extracellular pathogen-based immune responses and negatively regulated in response to intracellular pathogen-based responses such as viral infections. This theoretically should help decrease the number of HHV-8 susceptible cells in the host. We have shown that HHV-8 infected MDDC *in vitro* display a Th2-skewed cytokine response characterized by a significant increase in IL-6, MIP1α, and MIP1β expression within 72 h post-infection. Furthermore, while IL-12p40 increases, production of IL-12 p70 is significantly lower in infected MDDC compared to controls (Hensler et al., 2009). IL12 p70 production is necessary for the induction of an effective CTL response (Wang et al., 2001b). However, there is also evidence that DC derived from HIV-1 infected patients with KS retain the ability to prime CD8^+^ T cells against HHV-8 specific antigens (Stebbing et al., 2003), even though they may lack other functional abilities previously discussed.

Plasmacytoid dendritic cells are a subset of DC comprising less than 1% of PBMC that focus on recognizing and responding to viral infections via TLR signaling (West et al., 2011) and type 1 IFN production (Liu, 2005). HHV-8 can infect pDC resulting in cell activation and IFNα production as a result of TLR9 induced signaling prior to the production of a viral factor that reduces TLR9 signaling and subsequent IFNα production (West et al., 2011). The receptor by which HHV-8 infects pDC has not been identified. However, pDC precursors express DC-SIGN, indicating they could be susceptible to HHV-8 infection (Soilleux et al., 2002). Alternatively, uninfected DC at sites of infection could recognize and engulf distressed, lytically infected cells, such as apoptotic B cells, macrophages, and endothelial cells (Ueno et al., 2007). All together these sustained DC functions should contribute to minimizing HHV-8 pathogenesis and allowing otherwise healthy individuals to handle this chronic infection over a lifetime.

## CONCLUSION

The natural progression of HHV-8 infection of humans is not fully understood. However, it most commonly begins with entry though a mucosal surface, and likely spreads through the lymphatic system through which it accesses immune cells in mucosal associated lymphoid tissue and the circulatory system. The expression of viral cytokines, vGPCR, and microRNA homologs suggests that this virus goes to a great extent to alter host cellular activities in favor of viral replication, disease, and survival. It is, therefore, an enigma how humans deal with chronic life-long HHV-8 infections with minimal morbidity unless otherwise immunocompromised. It is likely that, as with most chronic viral infections, severe disease is not an endpoint goal of HHV-8 in healthy individuals. The virus likely hinders the pathogen detection and surveillance arm of the immune system sufficiently to remain hidden, possibly within B cells.

Despite a non-robust lytic replication cycle in DC, abortive HHV-8 infection is associated with dysfunctional maturation and a dampened T helper-1 response and up-regulation of T helper-2 cytokine production, yet such responses are sufficient to limit pathogenesis. Intracellular signaling through virus attachment alone may be enough to induce these effects and eliminate the pressure on the virus to establish lytic replication within DC, particularly if it can reproduce using other cells, such as B cells, macrophages, and endothelial cells without traumatic pathogenesis. This suggests HHV-8 intricately alters the immune response to prolong survival while conserving its surrounding environment, the host.

Human herpesvirus 8 infection of an immunocompromised host increases the likelihood of HHV-8 associated disease development, such as KS. In addition, immune suppression after HHV-8 infection due to, for example, secondary HIV-1 infection or organ transplantation, is also associated with disease development. We theorize, as others have in the past (Osman et al., 1999), that the dampened immune activation and specific HHV-8 responses controlling chronic HHV-8 infection in healthy persons are not sufficient in a significantly immune compromised environment. The tug-of-war between this virus and the human host appears sophisticated. However, the process has not been fully elucidated, and uncovering the components involved should continue to be of great interest to the fields of cancer biology, virology, immunology, and cell biology.

## Conflict of Interest Statement

The authors declare that the research was conducted in the absence of any commercial or financial relationships that could be construed as a potential conflict of interest.
